# Duration of observation required in detecting fasciculation potentials in amyotrophic lateral sclerosis using high-density surface EMG

**DOI:** 10.1186/1743-0003-9-78

**Published:** 2012-10-10

**Authors:** Ping Zhou, Xiaoyan Li, Faezeh Jahanmiri-Nezhad, William Zev Rymer, Paul E Barkhaus

**Affiliations:** 1 Sensory Motor Performance Program, Rehabilitation Institute of Chicago, 345 E. Superior St, Suite 1406, Chicago, IL 60611, USA; 2 Department of Physical Medicine and Rehabilitation, Northwestern University, Chicago, IL, USA; 3 Institute of Biomedical Engineering, University of Science and Technology of China, Hefei, China; 4 Department of Bioengineering, University of Illinois at Chicago, Chicago, IL, USA; 5 Department of Neurology, Medical College of Wisconsin and the Milwaukee Veterans Administration Medical Center, Milwaukee, WI, USA

## Abstract

**Background:**

High-density surface electromyography (HD-SEMG) has recently emerged as a potentially useful tool in the evaluation of amyotrophic lateral sclerosis (ALS). This study addresses a practical constraint that arises when applying HD-SEMG for supporting the diagnosis of ALS; specifically, how long the surface EMG should be recorded before one can be confident that fasciculation potentials (FPs) are absent in a muscle being tested.

**Methods:**

HD-SEMG recordings of 29 muscles from 11 ALS patients were analyzed. We used the distribution of intervals between FPs, and estimated the observation duration needed to record from one to five FPs with a probability approaching unity. Such an approach was previously tested by Mills with a concentric needle electrode.

**Results:**

We found that the duration of recording was up to 70 s in order to record a single FP with a probability approaching unity. Increasing recording time to 2 minutes, the probability of recording five FPs approached approximately 0.95.

**Conclusions:**

HD-SEMG appears to be a suitable method for capturing FPs comparable to intramuscular needle EMG.

## Introduction

Amyotrophic lateral sclerosis (ALS) is a chronic, progressive, degenerative disease of the upper and lower motor neurons that almost always results in reduced life expectancy. To date, no definite biologic marker exists to ascertain the diagnosis. To improve the diagnosis and ascertainment of ALS, Brooks et al. [1] developed consensus criteria using clinical data, imaging studies, and electrophysiological testing (i.e., electromyography [EMG]). While EMG does not make the diagnosis, it is an important adjunct test used to support the diagnosis.

Examination of spontaneous muscle activity is an important part of routine EMG, particularly in supporting the diagnosis of ALS and its variants [2,3]. Spontaneous EMG activity such as fibrillation potentials [2] and fasciculation potentials (FPs) are often seen, although other types of activity may also be recorded (e.g. complex repetitive discharges, myokymic discharges, neuromyotonic discharges, etc.). While fibrillation potentials are considered to be the *sine qua non* of active denervation such as seen in ALS, recent discussion has revisited the potential application of FPs to enhance the sensitivity of diagnosis of ALS [3-5]. In all cases of ALS, the routine EMG should also show enlarged, unstable, typically complex motor unit action potentials that represent ongoing reinnervation [6]. Though not unique or specific to ALS, it is this combination of EMG evidence for active denervation associated with evidence of chronic reinnervation that defines ALS electromyographically, in association with clinical criteria.

If new criteria are to be considered in the use of FPs to facilitate the earlier diagnosis of ALS [3,4], then it is reasonable to try to find ways to optimize their detection. During routine EMG examination of a patient for potential ALS, a practical consideration is: how long should we observe this needle electrode recording before concluding that fasciculation potentials are absent? [7]. Mills answered this question by calculating the probability values for observing one to five FPs in relation to duration of observation. It was found that using a needle electrode, up to 90 s may be required to record a single FP with a probability approaching unity while the duration of recording should be 180 s to observe five FPs [7].

Compared with positive sharp waves or fibrillation potentials that are detected solely by intramuscular needle electrodes, FPs can be recorded by both needle and surface electrodes [8]. In recent years, high-density surface EMG (HD-SEMG) techniques have been developed using electrode arrays comprised of a number of recording probes having minute skin-electrode contact area and small inter-electrode distances [9-12]. Because of the added spatial information, such electrode arrays can offer additional investigative and diagnostic components for examination of the neuromuscular system [13-19].

When clinicians use HD-SEMG to examine a patient with potential ALS, the analogous practical question similar to routine needle electrode EMG recordings is again “how long should the HD-SEMG signal be recorded before the tested muscle can be assumed not to have FPs?”. In this short report, we attempt to address this question using Mills’ approach by calculating the probability of detecting FPs in relation to observation duration of the HD-SEMG signal [7].

## Methods

Eleven subjects (8 males, 3 females, age: 57.4 ± 8.2 years, weight: 87.0 ± 26.6 kilograms, height: 175.5 ±10.9 cm) with Definite ALS or Probable ALS with Laboratory Support based on El Escorial criteria [1] participated in this study. All subjects gave written, informed consent prior to their participation. All data recordings were performed at the last author’s institution and had the approval of the local Human Studies Committee.

Each subject was positioned comfortably supine on an examination table with a pillow under their head. After placement of the surface electrode array for HD-SEMG signal detection, the subjects were asked to completely relax. The tested arm was placed in its natural, resting position. For the first dorsal interosseous (FDI) and thenar muscles, the hand was typically in semi-pronation. The biceps brachii (BB) was recorded with the elbow partially flexed and forearm in semi-pronation. HD-SEMG signals were recorded for at least 3 minutes in a relaxed condition. No feedback was provided to subjects during the recording.

**Figure 1:**
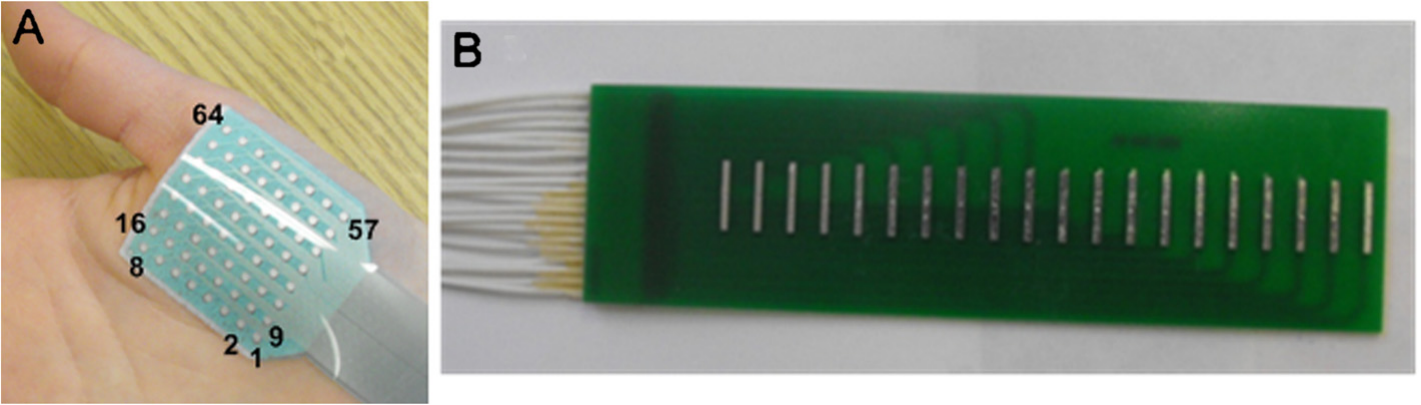
**The electrode arrays used for FP recordings.** (**A**) shows a flexible surface electrode array (TMS International BV, Enschede, The Netherlands) that contains 64 recording electrodes arranged in an 8 × 8 square matrix. The inter-electrode-distance (IED) between two consecutive recording electrodes in both (x and y) directions is 4 mm and each recording electrode has a circular recording surface of 1.2 mm in diameter. (**B**) shows our custom made 20-channel bar electrode array. The distance between two consecutive recording bars is 5 mm and each bar width is 1 mm in a linear arrangement.

In each experiment, a flexible surface electrode array (Figure 1A, TMS International BV, Enschede, The Netherlands) was used for FDI and thenar recordings. A 20-channel bar electrode array (Figure 1B, designed and fabricated in our laboratory) was used for BB recordings. The 2-dimensional flexible array covered the whole surface area of the FDI or thenar muscles while the linear bar electrode array was located from proximal to distal tendon junctions of the BB muscles. The surface electrode array signals were amplified by the Refa128 EMG Recording System (TMS International BV, Enschede, The Netherlands), with a reference electrode located on the olecranon (each channel also had a common feedback subtraction of the average of all the recording channels). The surface EMG signals were sampled at 2 kHz per channel, with a system band pass filter setting of 20–500 Hz. Before starting the recording, each channel’s baseline signal was carefully examined. If a channel’s signal quality was poor and further efforts failed to improve the signal, the channel was disconnected. It was typical that 2–5 channels were disconnected during the experiment. The quality of the signal was online monitored during data collection. Since the recording was performed in a resting muscle the signal quality usually remained stable.

The recorded HD-SEMG from the Refa128 system was imported to the Spike 2 software (version 5.12; Cambridge Electronics Design [CED], Cambridge, UK) for display and off-line processing. This software allows viewing or processing of selected channels of the electrode array and is useful for long duration and multi-channel signal processing. FPs were detected using a threshold-based spike detection algorithm across all the channels. The occurrence time of each FP from the start of the recording was determined, and was then imported to MatLab (Version R2008a, MathWorks, Natick, MA) for further processing.

For each recording, let *t* (1), *t* (2)…*t* (*n*), 1 ≤ *n* ≤ *N* represent the times of occurrence of FPs, and *N* is the total number of FPs. The intervals between FPs were calculated [7]:

(None) $$I\left(i\right)=t\left(n+i\right)-t\left(n\right),i=1,2,3,4,5;1\leq n\leq N-i$$

The maximum values of *I* (*i*) represent the longest intervals in which *i* FPs occurred. The cumulative frequency distribution of *I* (*i*) from the whole dataset was then constructed, allowing the probability of *i* FPs occurring with respect to the duration of the recording to be calculated. In other words, after the electrode array has been placed and recording started, what will be the time range before *i* FPs should have occurred?

## Results

**Figure 2:**
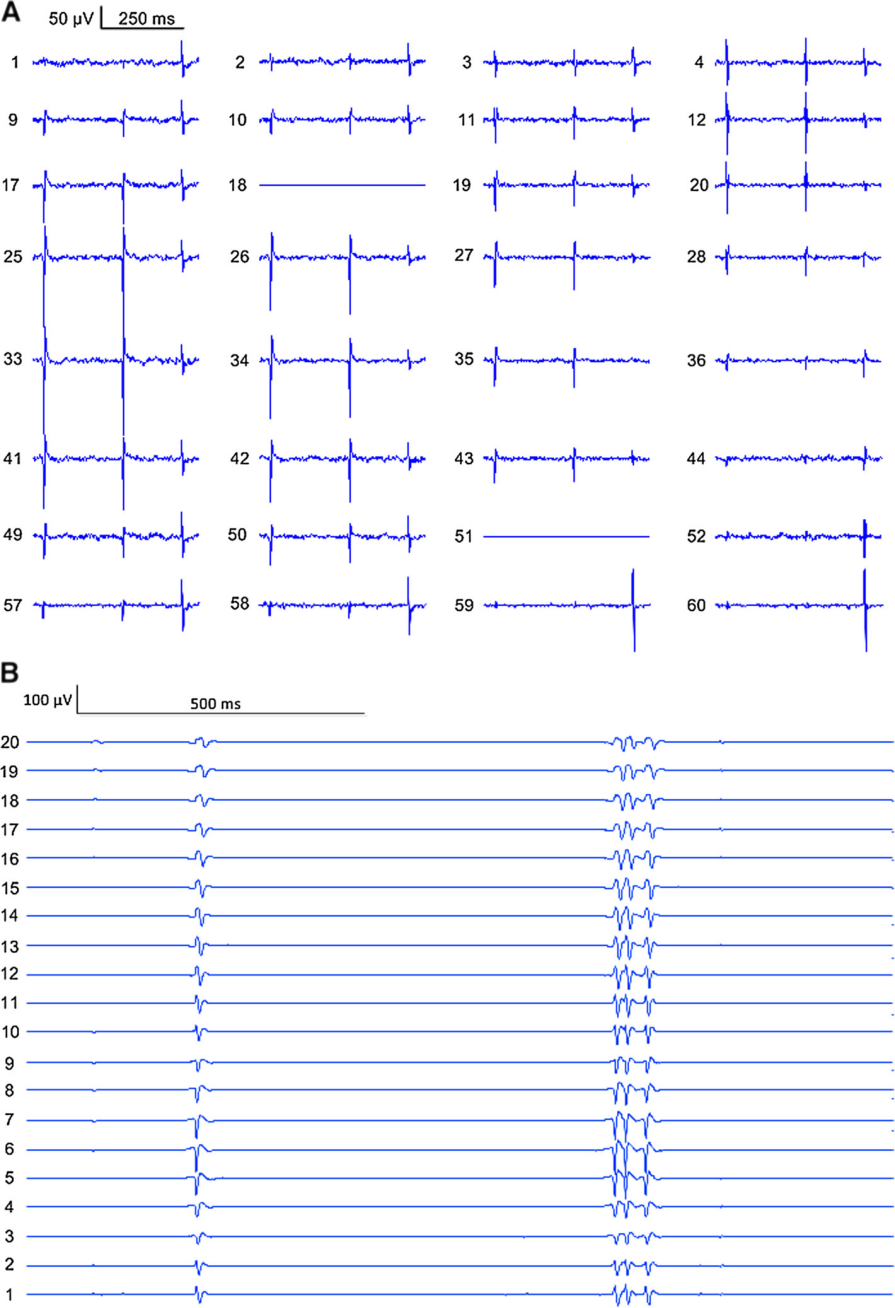
**Examples of surface EMG signals recorded using the two electrode arrays.** (**A**) Surface EMG signals recorded with the flexible electrode array from the thenar muscle. (**B**) Surface EMG signals recorded with the linear electrode array from the BB muscle. Channel 1 represents the most proximal channel.

**Figure 3:**
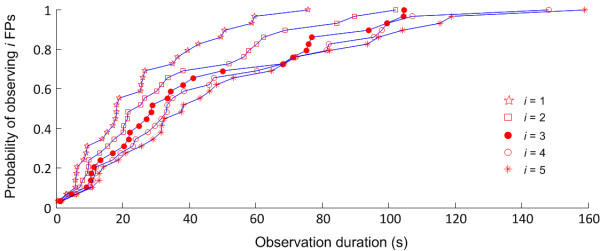
**Cumulative probability of observing *i *(*i*** **= 1,2,3,4,5, respectively) FPs with respect to observation duration of the HD-SEMG.**

The duration of each recording of spontaneous muscle activity from the ALS subjects varied from 215 s to 764 s. Figure 2 shows an example of FP recordings using the two different electrode arrays from the thenar and BB muscles, respectively. FPs were recorded from all tested muscles. The overall FP rate was 3.5-139.0 FPs/minute. The average skewness of the inter-FP-interval distribution was 1.06 ± 0.71, suggesting that the distribution of intervals between FPs was highly skewed with many short intervals and few long intervals. Across all muscles, the cumulative frequency distributions for one to five FPs are presented in Figure 3.

We show that the probability of recording a single FP using HD-SEMG was approximately 0.55 with a 20 s observation time and approximately 0.80 with a 40 s observation time. To record a single FP with a probability approaching unity, an observation time of up to 70 s may be needed. To record multiple FPs, a longer recording time is required as demonstrated in Figure 3. For example, the probability of recording five FPs approached approximately 0.95 with HD-SEMG recording for up to 2 minutes.

## Discussion

Intramuscular needle electrodes have been routinely used in the clinical EMG examination for evaluation and diagnosis of neuromuscular diseases [6]. In recent years, multi-channel recordings have been developed in surface EMG technology using electrode arrays up to 128 channels [9-13]. Indeed, development of the multielectrode surface EMG montage represents an important research focus in several recent collaborative efforts sponsored by European Community (EC) [20]. HD-SEMG has been emerging as a potential clinical tool to supplement routine needle EMG. The spatial information of the electrode array can provide additional useful information (e.g., muscle fiber conduction velocity, motor unit territory, innervation zone localization) which is impractical or not possible to obtain with conventional needle electrodes [13-19].

For some specific evaluations, HD-SEMG may provide a potential substitute for routine needle EMG, while preserving diagnostic sensitivity and specificity. One example is to record FPs as demonstrated in this short report, as well as in previous studies [21-23]. Fasciculations are a characteristic and potentially diagnostic feature of ALS [24-27], although their precise role in establishing the diagnosis of ALS remains contentious based on routine needle EMG [3,4]. FPs may portend lower motoneuron dysfunction prior to clinical symptoms such as weakness or muscle atrophy, possibly before the onset of definite reinnervation changes in the motor unit action potentials [6,22,24,25]. Thus, enhanced detection of FPs may be of great importance for early diagnosis of ALS as well as better understanding their pathophysiology in this disorder.

In this brief report, we address the practical question of how long HD-SEMG should be used to record the tested muscle before being confident that no FPs are present. The method proposed by Mills [7] for testing with an intramuscular needle EMG electrode was utilized in this study. Considering the practicality in time needed for needle electrode recordings, Mills used a one minute recording epoch as the criterion in how long to monitor a muscle for FPs before it can be included in analysis. In other words, the muscle was excluded from analysis if it showed no FPs after 1 minute. In this study, up to 764 s spontaneous EMG signal was recorded for each muscle. These longer recording times avoided exclusion of muscles with long inter-fasciculation intervals. Hence we were able to calculate the probability function of observing one to five FPs with respect to recording duration. Although with a 2-dimensional surface electrode array it is feasible to differentiate FPs arising from different motor units using their waveform morphology to identify them with the added spatial resolution (particularly in perpendicular to muscle fibers) [28,29], our intent was to determine the longest interval between FPs regardless of waveform since these would have the same diagnostic significance. Our results confirm that HD-SEMG offers a suitable and highly sensitive technique for capturing FPs. We acknowledge that simultaneous recordings with needle EMG and HD-SEMG from the same muscles would be necessary to directly compare sensitivity in FP detection between them. In addition, this study tested patients with Definite ALS or Probable ALS with Laboratory Support. It is presently unknown how the results may change for patients with Possible ALS or Suspected ALS.

It should be noted that the prediction of time for EMG surveillance in detecting FPs depends on the context of clinical situation [7]. With clear conventional evidence of denervation (i.e., fibrillation potentials) in a muscle, identification of a single FP may be sufficient to further support the possibility of ALS. Thus the longest time needed to observe such a muscle using HD-SEMG would be approximately 70 s. Conversely, when clinical and conventional EMG abnormality is limited, a longer duration recording may be necessary. In this situation, the detection of FPs could have greater significance in determining electrophysiologic abnormality in a muscle. The current analysis demonstrates the longest time to be certain of recording one to five FPs using HD-SEMG. In most cases, however, FPs may be detected more quickly. After the suggested maximum recording time (approximately 70s for 1 FP; 120 s for 5 FPs), we feel confident that FPs are very likely to be absent in a tested muscle.

## Competing interests

The authors declare that they have no competing interests.

## Authors’ contributions

PZ performed experiment design, data collection and analysis, interpretation of the results, and drafting of the manuscript. XL was involved in data collection and analysis. FJ was involved in data collection. WR was involved in interpretation of the results and revision of the manuscript. PB was involved in ALS subject recruitment, interpretation of the results, and revision of the manuscript. All authors read and approved the final manuscript.
